# Gradual reduction in exercise capacity in chronic kidney disease is associated with systemic oxygen delivery factors

**DOI:** 10.1371/journal.pone.0209325

**Published:** 2018-12-19

**Authors:** Helena Wallin, Anna M. Asp, Carin Wallquist, Eva Jansson, Kenneth Caidahl, Britta Hylander Rössner, Stefan H. Jacobson, Anette Rickenlund, Maria J. Eriksson

**Affiliations:** 1 Department of Laboratory Medicine, Division of Clinical Physiology, Karolinska Institutet, Stockholm, Sweden; 2 Department of Clinical Physiology, Karolinska University Hospital, Stockholm, Sweden; 3 Department of Molecular Medicine and Surgery, Karolinska Institutet, Stockholm, Sweden; 4 Department of Nephrology, Karolinska University Hospital, Karolinska Institutet, Stockholm, Sweden; 5 Department of Molecular and Clinical Medicine, Institute of Medicine, Sahlgrenska Academy, University of Gothenburg, Gothenburg, Sweden; 6 Division of Nephrology, Department of Clinical Sciences, Karolinska Institutet Danderyd University Hospital, Stockholm, Sweden; International University of Health and Welfare, School of Medicine, JAPAN

## Abstract

**Background:**

The cause of reduced exercise capacity (ExCap) in chronic kidney disease (CKD) is multifactorial. The aim of this study was to investigate determinants of aerobic ExCap in patients with mild to severe CKD not undergoing dialysis.

**Methods:**

We included 52 individuals with CKD stage 2–3, 47 with stage 4–5, and 54 healthy controls. Peak workload and peak heart rate (HR) were assessed by a maximal cycle exercise test. Cardiac function including stroke volume (SV) and vascular stiffness were evaluated by ultrasound at rest. Handgrip strength, body composition, haemoglobin level and self-reported physical activity were assessed.

**Results:**

Peak workload (221±60, 185±59, 150±54 W for controls, CKD 2–3 and CKD 4–5 respectively), peak HR (177±11, 161±24, 144±31 beats/min) and haemoglobin level (14.2±1.2, 13.5±1.4, 12.2±1.3 g/dL) were all three significantly lower in CKD 2–3 than in controls, (p = 0.001, 0.001 and 0.03 respectively) and were even lower in stages 4–5 CKD than in CKD 2–3 (p = 0.01, 0.001 and <0.001 respectively). Resting SV and lean body mass did not differ between groups and handgrip strength was significantly lower only in CKD 4–5 compared to controls (p = 0.02). Peak workload was strongly associated with the systemic oxygen delivery factors: SV, peak HR and haemoglobin level. These three factors along with age, sex and height^2^ explained 82% of variation in peak workload. Peak HR contributed most to the variation; the peripheral variables handgrip strength and vascular stiffness did not improve the explanatory value in regression analysis.

**Conclusions:**

In this cross-sectional study of CKD patients not on dialysis, aerobic ExCap decreased gradually with disease severity. ExCap was associated mainly with systemic oxygen delivery factors, in particular peak HR. Neither muscle function and mass, nor vascular stiffness were independent determinants of aerobic ExCap in this group of CKD patients.

## Introduction

Reduced aerobic exercise capacity (ExCap) is a well-established characteristic of end-stage renal disease [1–3] and there are also reports of reduced ExCap in early stages of chronic kidney disease (CKD) [4,5]. Importantly, reduced physical function has a negative impact on daily life activities and is associated with increased mortality in individuals with CKD [3,6,7]. Understanding the causes of this decreased ExCap early in the disease process may help to improve care for individuals with CKD. Peak workload on cycle ergometer and peak oxygen uptake (VO_2_peak) are linearly and closely related and can both be used as measurements of ExCap [8].

Aerobic ExCap is determined by maximal oxygen uptake (VO_2_max), which is a function of oxygen delivery and oxygen utilization [9]. Systemic oxygen delivery is determined mainly by stroke volume (SV), maximal heart rate (HR) and haemoglobin concentration, whereas peripheral oxygen delivery is influenced by factors such as capillary blood flow and oxygen diffusion from the blood to muscle mitochondria [10]. The systemic and peripheral factors are likely to be interdependent [11].

In young healthy people, individual variation in aerobic ExCap and VO_2_max is determined primarily by differences in systemic oxygen delivery, in particular by differences in SV [10–12]. By contrast, in CKD, individual variation in aerobic ExCap and VO_2_max may be determined by other factors. In both dialysis-dependent and non-dialysis CKD, peak HR [2, 4, 13, 14] and haemoglobin level [2, 4, 15, 16] are reduced. A few studies have reported that reduced oxygen delivery because of low peak HR and haemoglobin level are associated with and may limit VO_2_peak in both patient groups [2, 4, 13].

In addition to the systemic factors, peripheral factors such as dysfunctional microcirculation, mitochondrial dysfunction, and loss of muscle mass may contribute to the decline in aerobic ExCap and VO_2_max in patients on dialysis. For example, isokinetic muscle strength was a better predictor of VO_2_peak than blood oxygen carrying capacity in a study by Diesel et al [17] and improvement in VO_2_peak after training was mainly attributable to a widened arteriovenous oxygen difference in another group of dialysis patients [18]. While changes in markers of muscle mass and strength and in the muscle architecture are well-known features of dialysis-dependent CKD [19–21], peripheral involvement is less studied in non-dialysis CKD, but may nonetheless be of importance. There is some evidence of reduced handgrip strength [22] and histopathological changes in the skeletal muscle [23, 24] in this population. Moreover, vascular stiffness, which may influence the microcirculation and blood flow distribution [25], has been associated with reduced ExCap in non-dialysis CKD [5].

In summary, the mechanisms behind reduced ExCap and VO_2_peak have been extensively explored in dialysis patients, and attributed to changes in both systemic oxygen delivery and peripheral factors. The impact of peripheral mechanisms on aerobic ExCap has been scarcely studied in non-dialysis CKD and the combined impact of both systemic and peripheral mechanisms even less. Therefore, the aim of this study was to evaluate the importance of both systemic oxygen delivery factors and peripheral factors for aerobic ExCap in mild-to-severe non-dialysis CKD. Handgrip strength, lean body mass (LBM) and vascular stiffness represent peripheral factors in the present study.

## Methods

The current analyses are part of a larger, prospective, single-centre cohort study, PROGRESS 2002 on factors impacting progress of renal insufficiency, the details of which have been described previously [26].

### Study protocol

Ninety-nine patients with non-dialysis CKD (aged 18–65 years) and 54 healthy controls who had completed an exercise test were included (Table 1). Glomerular filtration rate (GFR) was measured by iohexol plasma clearance [27]. Patients were recruited consecutively from the outpatient clinic at the Department of Renal Medicine at the Karolinska University Hospital during 2002–2009 if they had renal function corresponding to CKD stages 2–3 ((52 patients with GFR 60 ± 5.2 mL/min/1.73 m^2^) or CKD stages 4–5 (47 patients with GFR 15 ± 3.8 mL/min/1.73 m^2^) as defined by the National Kidney Foundation [28]. 54 controls (GFR 99 ± 13 mL/min/1.73 m^2^), matched for age and sex with the CKD 2–3 group, were randomly selected from the Swedish Total Population Register or recruited through the website of the regional university hospital. The exclusion criteria for all participants were current malignancy, kidney transplantation or kidney donation, or blood-transmitted disease. The inclusion criteria for the controls were absence of kidney disease, cardiovascular disease, diabetes or any chronic medication. After inclusion, all participants underwent clinical investigation, anthropometric measurement, exercise stress test, handgrip strength test, laboratory testing, carotid ultrasound, transthoracic echocardiography and body composition scan.

**Table 1:** Study group characteristics.

Variables	Controls	CKD 2–3	CKD 4–5	p-value		p-value post hoc
Subjects (n)	54	52	47			
Age (years)	48 ± 11	47 ± 11	49 ± 12	0.7		
Male, n (%)	35 (65)	32 (62)	28 (60)	0.9		
Height (cm)	175 ± 9	174 ± 9	174 ± 10	0.6		
Weight (kg)	77 ± 12	76 ± 16	77 ± 17	0.9		
BMI (kg/m^2^)	24.8 ± 3.4	25.1 ± 4.0	25.4 ± 4.1	0.8		
BSA (m^2^)	1.92 ± 0.19	1.92 ± 0.21	1.91 ± 0.24	0.9		
Lean body mass (kg)	54 ± 11	52 ± 11	51 ± 11	0.5		
Body fat (%)	24 ± 8	25 ± 8	27 ± 9	0.1		
Ever smoker, n (%)	20 (38)	26 (51)	25 (54)	0.2		
Current smoker	6 (11)	7 (14)	7 (15)	0.9		
Diabetes		10 (19)	7 (15)	0.6		
**Etiology of CKD, n (%)**						
Familial/hereditary/congenital disease		14 (27)	16 (34)	0.9		
Primary glomerulonephritis		17 (33)	9 (19)	0.1		
Secondary glomerular/systemic disease		9 (17)	8 (17)	0.8		
Miscellaneous/unknown		12 (23)	14 (30)	0.5		
**Medication, n (%)**						
Beta blocker		10 (19)	17 (36)	0.06		
Diuretics		12 (23)	32 (70)	<0.001		
ACE inhibitors		22 (42)	28 (60)	0.09		
Angiotensin II blockers		21 (39)	22 (47)	0.4		
Calcium-channel blockers		10 (19)	28 (60)	<0.001		
Lipid-lowering therapy		13 (25)	31 (66)	<0.001		
**Muscular function**						
Handgrip strength (kg)	44 ± 12	40 ± 11	38 ± 14	0.02	0.2^a^/0.02^b^/0.6^c^	
**Cardiac structure and function**						
LVED (cm)	4.7 ± 0.5	4.7 ± 0.6	4.6 ± 0.6	0.8		
LVMI (g/m^2^)	91 ± 18	99 ± 30	107 ± 28	0.008	0.2^a^/0.006^b^/0.3^b^	
LV ejection fraction, EF (%)	66 ± 8	62 ± 8	61 ± 10	0.04	0.08^a^/0.06^b^/0.9^c^	
LV diastolic function, E/é	5.0 ± 1.3	5.7 ± 1.5	6.4 ± 1.8	<0.001	0.07^a^/<0.001^b^/0.07^c^	
Stroke volume (mL)*	72 ± 16	66 ± 17	66 ± 14	0.1		
Stroke volume index (mL/m^2^)	37 ± 7	34 ± 8	35 ± 6	0.06		
RV systolic function, TAPSE (cm)	2.5 ± 0.5	2.3 ± 0.5	2.2 ± 0.4	0.03	0.2^a^/0.03^b^/0.7^c^	
**Vascular function**						
Vascular stiffness, Ep (N/m^2^) × 10^4^	6.1 (4.8–6.9)	6.5 (5.4–8.1)	7.4 (5.2–9.9)	0.01	0.2^a^/0.01^b^/0.9^c^	
**Laboratory measurements**						
GFR (mL/min/1.73 m^2^)	99 ± 12	60 ± 5.2	15 ± 3.8	<0.001	<0.001 for all	
Haemoglobin (g/dL)	14.2 ± 1.2	13.5 ± 1.4	12.2 ± 1.3	<0.001	0.03^a^/<0.001^b^/<0.001^c^	
*Patients not taking beta blockers* (n)	52	41	24			
Stroke volume (mL)	72 ± 16	66 ± 18	61 ± 12	0.02	0.2^a^/0.02^b^/0.4^c^	
Stroke volume index (mL/m^2^)	37 ± 7	35 ± 8	33 ± 5	0.04	0.2^a^/0.04^b^/0.6^c^	

Values reported as number (percentage) or mean ± standard deviation or median (interquartile range) for skewed variables.

p-values are for between-groups (Tukey honest significant post hoc test)

^a^CKD 2–3 vs controls

^b^CKD 4–5 vs controls

^c^CKD 2–3 vs CKD 4–5.

n–number of subjects and indicates the maximal number of subjects per group. Because of missing values the total number for each of the variables varies between 142 and 153 with most missing values for stroke volume.

*n = 52/51/39.

BMI–body mass index, LVED–left ventricular end-diastolic diameter, LVMI–left ventricular mass index, LV–left ventricle, E/é–early filling velocity/early diastolic myocardial velocity, RV–right ventricle, TAPSE–tricuspid annular plane systolic excursion, Ep–pressure strain elastic modulus in the carotid artery, GFR–glomerular filtration rate

The study protocol was reviewed and approved by the Local Ethics Committee and Institutional Review Board of the Karolinska Institutet at the Karolinska University Hospital. All participants gave their written informed consent.

### Aerobic exercise capacity

A symptom-limited incremental cycle ergometer test on an electronically braked cycle ergometer (RE990, Rodby Innovation AB, Uppsala, Sweden) was administered according to clinical standards. The initial workload and workload increase/minute (10, 15 or 20 W) were individualized with the goal of achieving symptom limitation within 6 to 10 minutes. Participants were instructed to cycle at a speed of 60 rpm and were encouraged to continue cycling until exhaustion. Perceived exertion was reported as the highest rating (from 1 to 10 on the Borg CR10 scale [29] for leg fatigue, dyspnoea or general exhaustion as a limiting symptom. Aerobic ExCap was defined as the peak workload in W; however, because different ramps were used, the value was adjusted to 10-W increments for women and 15-W increments for men [30]. Resting HR and blood pressure (BP) were measured in the supine position before the exercise test. BP was measured using a sphygmomanometer. Peak systolic BP was defined as the last value measured before the end of exercise. Continuous 12-lead electrocardiography was used to measure HR and for ST–T-segment monitoring and safety purposes. Predicted values for normal ExCap were derived from a Swedish population study that takes into account age, sex, height and workload increment per minute [31]. Predicted peak HR was calculated as 220 minus age. Heart rate reserve (HR reserve) was defined as the difference between peak HR and resting HR. HR recovery (HR recovery) was defined as the difference between peak HR and HR at 1 and 2 min after the end of the exercise test.

### Muscular function

A handheld Takei^TM^ dynamometer (Grip-A; Takei Scientific Instruments Co., Ltd, Tokyo, Japan) was used to measure maximum voluntary isometric contraction as a measure of handgrip strength. The test was performed with the participant in a standing position. The measurement was repeated three times using the dominant arm, and the highest value was recorded as handgrip strength.

### Cardiac and vascular function

Echocardiography was performed only at rest, with a patient in the supine left decubitus position and according to current guidelines [32]. The images were acquired using an ultrasound scanner (Sequoia 512; Siemens Medical Solutions, Mountain View, CA, USA) with an appropriate transducer. Resting SV was calculated as the difference between left ventricular (LV) diastolic and systolic volumes. In some participants, SV could not be calculated because of suboptimal image quality. LV mass and SV were indexed for body surface area (SVI). To estimate LV diastolic function, the ratio between the early mitral flow velocity (E) and the averaged septal and lateral early diastolic myocardial velocity (é) was used. Right ventricular systolic longitudinal function was expressed as the tricuspid annular plane systolic excursion (TAPSE). Carotid artery ultrasound was performed to assess arterial stiffness. Measurements of the diameter of the right common carotid artery and calculations of the pressure strain elastic modulus (Ep) were performed according to a standardized protocol [33].

### Body composition

Body composition was measured with a whole-body dual-energy X-ray absorptiometry scan to determine LBM and body fat percentage (Hologic QDR 4500 Discovery A, Bedford, MA, USA; software version 12.3).

### Physical activity level

Physical activity level was rated by the participants using a four-point scale modified from the Saltin–Grimby Physical Activity Level Scale [34] as follows.

Level 1 = *Regular exercise*: running, swimming, tennis, badminton, gymnastics or similar activity on three or more occasions per week; every session should last at least 30 min and cause sweating.

Level 2 = *Moderate amount of regular exercise*: running, swimming, tennis, badminton, gymnastics or similar activity on 1–2 occasions per week; every session should last at least 30 minutes and cause sweating.

Level 3 = *Light exercise*: walking or cycling or other physical activity during at least 2 h per week, usually without sweating; this includes walking or cycling to/from work, Sunday walks, gardening, fishing, table tennis and bowling or similar activity.

Level 4 = *Sedentary*: mostly reading, watching television, movies or other sedentary activities, or walking, cycling or light exercise for less than 2 h per week.

### Blood samples

Haemoglobin concentration was measured in venous blood obtained using routine laboratory methods at the Karolinska University Laboratory at the inclusion visit before the exercise test.

### Calculations

The rate of systemic oxygen delivery (cardiac output x oxygen content) during peak exercise, expressed as millilitres of oxygen per minute, was estimated by the following equation:

Peak systemic oxygen delivery = peak SV × peak HR x ((1.34 × haemoglobin concentration × oxygen saturation) + (0.03 × oxygen tension)).

Assuming a normal blood oxygen saturation of 100% and a normal blood oxygen tension of 100 mmHg, the equation may be simplified as follows:

Peak systemic oxygen delivery = peak SV × peak HR × (1.34 × haemoglobin concentration + 3).

The factor of 1.34 equals the oxygen-binding capacity of haemoglobin, expressed as millilitres of oxygen per gram of haemoglobin (per g/L blood). The factor of 0.03 equals the oxygen solubility coefficient, expressed as millilitres of oxygen per litre of blood at an oxygen tension of 100 mmHg.

SV measured at rest in the supine position was used as an approximation of peak SV in the calculation [35, 36].

### Statistical analysis

Continuous variables are presented as mean and standard deviation, and categorical variables as number and percentage of the study sample. Skewed variables are presented as median and interquartile range. Logarithmic transformation was used for skewed variables when analysed in multiple regression models.

Group comparisons were performed using one-way analysis of variance, with Tukey’s honest significant difference post hoc test for further between-group analysis; Kruskal–Wallis or chi-square tests were used when appropriate. Bonferroni post hoc testing was performed to adjust p-values between groups when the Kruskal–Wallis test was used. For relevant variables, subgroup analysis was performed in patients not taking beta-blockers. Statistical significance was defined as a p-value <0.05 for a two-tailed test. Partial correlations were calculated to investigate the relationships between two variables while controlling for the influence of a third variable.

To explore the determinants of aerobic ExCap in CKD, the two CKD groups were merged into one group (CKD stage 2–5). Peak workload was used as the dependent variable in multiple linear regression analyses following four different strategies (1–4 below). Standardized regression coefficients (RCs) are reported. Adjustment for age, sex and height squared was performed in all analyses because these have been identified as non-disease-related determinants of peak workload in reference materials [8, 31]. Height squared is consequently abbreviated to height in the text.

1. **Single determinants—**The increase in the explanatory value (R^2^) for peak workload was analysed after adding single variables to a model with age, sex and height in both CKD 2–5 and controls. This purpose of this analysis was to better understand the contribution of individual variables to ExCap in CKD compared to controls.
2. **Multiple determinants**—The multiple regression analysis performed in CKD 2–5 with peak workload as the dependent variable included the systemic oxygen delivery factors (SV, peak HR and haemoglobin level) and the peripheral factors (handgrip strength and vascular stiffness) after adjustment for age, sex and height.
3. **Stepwise multiple determinants—**Manual forward regression was used to analyse the stepwise increase in R^2^ for peak workload in CKD 2–5 by adding the significant independent variables from the regression analysis described above (*multiple determinants*) after adjustment for age, sex and height.

Statistical analyses were performed using IBM SPSS Statistics for Windows (version 23.0; IBM, Armonk, NY, USA).

## Results

The clinical characteristics for each group (controls, CKD 2–3 and CKD 4–5) are presented in Table 1. Handgrip strength was significantly lower only in CKD 4–5 compared to controls, while there was no significant difference between CKD 2–3 and controls. The same trend was seen in vascular stiffness, which was significantly higher only in CKD 4–5, but not in CKD 2–3, compared to controls. LBM did not differ significantly between groups. Self-reported physical activity was significantly lower in CKD 4–5 than in controls (Table 2).

**Table 2:** Self-reported physical activity level.

	Controls	CKD 2–3	CKD 4–5
Subjects (n)	54	52	47
PA level 1	14 (26)	10 (19)	4 (8)
PA level 2	21 (39)	15 (29)	9 (19)
PA level 3	19 (35)	19 (37)	29 (62)
PA level 4	0	8 (15)	5 (11)

Values reported as number (percentage). n–number of subjects; PA–physical activity

p-value between all groups <0.001 (Kruskal–Wallis). p-values between groups after Bonferroni correction: 0.08 (CKD 2–3 vs controls); <0.001 (CKD 4–5 vs controls); 0.3 (CKD 2–3 vs CKD 4–5).

### Exercise capacity and oxygen delivery factors

Peak workload, exercise time, peak HR, HR reserve, estimated peak systemic oxygen delivery and HR recovery at 2 min after the end of exercise were all significantly lower in CKD 2–3 than in controls, and these values were even lower in CKD 4–5 (Table 3). A similar pattern was found when analysing CKD subgroups not taking beta-blockers compared with controls as well as subgroups without diabetes. All patients exercised until near exhaustion, and the rating of perceived exertion did not differ between groups. Haemoglobin level was significantly lower in CKD than in controls and decreased progressively with CKD severity (i.e., lower in CKD 4–5 than in CKD 2–3) (Table 1). SV and SVI did not differ significantly between the study groups. By contrast, in CKD subgroups not taking beta-blockers, SV and SVI were significantly lower in CKD 4–5 than in controls (Table 1).

**Table 3:** Response to exercise.

Variables	Controls	CKD 2–3	CKD 4–5	p-value	p-value post hoc
Subjects (n)	54	52	47		
**Rest**					
HR (bpm)	70 ± 10	70 ± 12	72 ± 14	0.6	
SBP (mmHg)	125 ± 14	132 ± 20	138 ± 23	0.003	0.2^a^/<0.001^b^/0.2^c^
DBP (mmHg)	78 ± 9	78 ± 9	80 ± 9	0.3	
**Exercise**					
Peak workload (W)	221 ± 60	185 ± 59	150 ± 54	<0.001	0.001^a^/<0.001^b^/0.01^c^
Exercise time (min)	10.5 ± 2.3	9.3 ± 2.1	7.9 ± 2.3	<0.001	0.01^a^/<0.001^b^/0.01^c^
Peak workload/predicted workload (%)	104 ± 18	88 ± 19	73 ± 16	<0.001	<0.001 for all
Peak RPE	8.9 ± 2	9.0 ± 1	8.9 ± 1	0.9	
Peak HR (bpm)	177 ± 11	161 ± 24	144 ± 31	<0.001	0.001^a^/<0.001^b^/0.001^c^
Peak HR/predicted HR (%)	103 ± 7	93 ± 12	84 ± 16	<0.001	<0.001^a^/<0.001^b^/0.001^c^
HR reserve (bpm)	107 ± 11	90 ± 26	72 ± 25	<0.001	<0.001 for all
HR recovery 1 min (bpm)	28 ± 8	27 ± 12	20 ± 9	<0.001	0.7^a^/<0.001^b^/0.001^c^
HR recovery 2 min (bpm)	59 ± 10	53 ± 14	45 ± 11	<0.001	0.008^a^/<0.001^b^/<0.001^c^
Peak SBP (mmHg)	195 ± 25	198 ± 34	186 ± 27	0.1	
Peak systemic O_2_ delivery (mL/min)*	2450 ± 627	1958 ± 653	1571 ± 402	<0.001	<0.001^a^/<0.001^b^/0.006^c^
*Patients not taking beta-blockers (n)*	54	42	30		
Peak workload (W)	221 ± 60	188 ± 62	151 ± 56	<0.001	0.02^a^/<0.001^b^/0.03^c^
Peak HR/predicted HR (%)	103 ± 7	96 ± 9	91 ± 9	<0.001	<0.001^a^/<0.001^b^/0.04^c^
HR reserve (bpm)	107 ± 11	96 ± 21	81 ± 19	<0.001	0.006^a^/<0.001^b^/0.001^c^
Peak systemic O_2_ delivery (mL/min)^€^	2450 ± 627	2018 ± 676	1625 ± 394	<0.001	0.003^a^/<0.001^b^/0.04^c^
*Patients without diabetes mellitus (n)*	54	42	40		
*Peak workload*	221±60	192±57	154±53	<0.001	0.04^2^/<0.001^b^/0.009^c^
*Peak HR/predicted HR*	103±7	93±11	86±15	<0.001	<0.001^2^/<0.001^b^/0.006^c^

Values reported as number ± standard deviation. p-values between groups (Tukey honest significant difference post hoc test)

^a^CKD 2–3 vs controls

^b^CKD 4–5 vs controls and

^c^CKD 2–3 vs CKD 4–5.

n–number of subjects; HR–heart rate; bpm–beats per minute; SBP–systolic blood pressure; DBP–diastolic blood pressure; Predicted workload–predicted peak workload according to reference formula; RPE–rating of perceived exertion; Predicted HR–age-predicted maximal HR; HR reserve–difference between peak HR and resting HR; O_2_ –oxygen.

*Calculated as described in methods section for 52 controls, 51 CKD 2–3 patients and 39 CKD 4–5 patients.

^€^Calculated as described in methods section for 52 controls, 41 CKD 2–3 patients and 24 CKD 4–5 patients.

### Determinants of aerobic exercise capacity

Tested as single determinants, the systemic oxygen delivery factors (SV, peak HR and haemoglobin level), HR reserve, E/é, vascular stiffness, LBM, handgrip strength and physical activity level were all associated with peak workload in CKD 2–5 after adjusting for age, sex and height (Table 4). Among these variables, peak HR, HR reserve and physical activity had the strongest effect sizes (RC value) and increased R^2^ the most. In the controls, only SV (but not peak HR or haemoglobin level) was associated with peak workload after adjusting for age, sex and height. LBM, handgrip strength and physical activity were also associated with peak workload in the controls.

**Table 4:** Determinants of aerobic exercise capacity (peak workload) in CKD 2–5 and controls identified by multiple regression analysis.

	CKD 2–5				Controls			
Variable	R^2^/R^2^^§^	RC	p	n	R^2^/R^2^^§^	RC	p	n
Age, sex, height ^2^	0.5			99	0.68			54
Lean body mass	0.49/0.52	0.41	0.02	96	0.68/0.75	0.56	<0.001	
Diabetes	0.50/0.52	–0.16	0.03	99	NA	NA	NA	
Handgrip strength	0.51/0.58	0.53	<0.001	97	0.68/0.71	0.33	0.03	54
Stroke volume	0.53/0.60	0.33	<0.001	90	0.71/0.77	0.33	0.001	52
E/é	0.55/0.58	–0.18	0.02	95	0.68/0.69	0.09	0.4	53
Ep^‡^	0.50/0.53	–0.22	0.02	98	0.68/0.68	0.02	0.8	54
GFR	0.50/0.56	0.26	<0.001	99	0.68/0.69	0.05	0.6	54
Haemoglobin	0.50/0.55	0.21	0.004	99	0.68/0.68	0.02	0.9	54
Resting HR	0.50/0.55	–0.23	0.001	99	0.68/0.68	–0.01	0.9	54
Peak HR	0.50/0.67	0.50	<0.001	99	0.68/0.69	0.11	0.2	54
Peak HR/predicted HR	0.50/0.67	0.43	<0.001	99	0.68/0.69	0.12	0.2	54
HR reserve	0.50/0.79	0.62	<0.001	99	0.68/0.69	0.12	0.2	54
Peak HR without bb	0.56/0.65	0.42	<0.001	72	NA	NA	NA	
HR reserve without bb	0.56/0.79	0.56	<0.001	72	NA	NA	NA	
PA level	0.50/0.65		<0.001^£^	99	0.68/0.74		0.01^£^	54
PA level 2^∞^		–0.16	0.08			–0.22	0.03	
PA level 3^∞^		–0.47	<0.001			–0.31	0.004	
PA level 4^∞^		–0.40	<0.001			NA	NA	

Each analysis included the non-disease-related variables age, sex and height^2^, and one additional variable. R^2^ –R^2^ for model with age; sex and height^2^

^§^R^2^ –R^2^ for model with age, sex and height^2^ with the addition of the specified variable.

^‡^lnEp.

^∞^Compared with PA level 1.

^£^p-value for F change from model with only age, sex and height^2^.

n–number of subjects in regression analysis for specific variable; RC–standardized regression coefficient for specified variable; NA–not applicable; E/é–early filling velocity/early diastolic myocardial velocity; Ep–pressure strain elastic modulus in the carotid artery; GFR–glomerular filtration rate; HR–heart rate; Predicted HR–age-predicted maximal HR; HR reserve–difference between peak HR and resting HR; without bb–only patients who were not taking beta-blocking therapy were included in the analysis; PA–physical activity

Tested as multiple determinants, in CKD 2–5 after adjusting for age, sex and height, systemic oxygen delivery factors (SV, peak HR and haemoglobin level) contributed significantly to explain the individual variation in peak workload (Table 5). Vascular stiffness and handgrip strength did not contribute significantly to the individual variation in peak workload. After adjusting for age in CKD 2–5, a significant correlation was found between handgrip strength and peak HR (r = 0.29, p = 0.004) but the correlation was not significant in controls (r = –0.01, p = 0.9). This correlation may have contributed to the lack of the separate effects of handgrip strength to the multiple regression results described above. After adjusting for age in CKD 2–5, vascular stiffness (Ep) and peak HR did not correlate significantly (r = –0.10, p = 0.3). Handgrip strength and LBM correlated significantly in CKD 2–5 (r = 0.8, p < 0.001), and therefore, only one of these measures was included in the multiple regression analyses.

**Table 5:** Determinants of aerobic exercise capacity (peak workload) in CKD 2–5 identified by multivariate regression analysis in 87 subjects*.

Variables	RC	p-value
Age	0.02	0.8
Male sex	0.13	0.2
Height^2^	0.14	0.1
Stroke volume	0.44	<0.001
Peak HR	0.41	<0.001
Haemoglobin	0.18	0.001
Handgrip strength	0.15	0.1
Ep^‡^	–0.10	0.09

The overall R^2^ for the model was 0.83. R^2^ –explanatory value of the model; RC–standardized regression coefficient; HR–heart rate; Ep–pressure strain elastic modulus; E/é–early filling velocity/early diastolic myocardial velocity

^‡^lnEp–Ep expressed as natural logarithm.

*n = 85 because of missing values in one or more variables.

**Fig 1:**
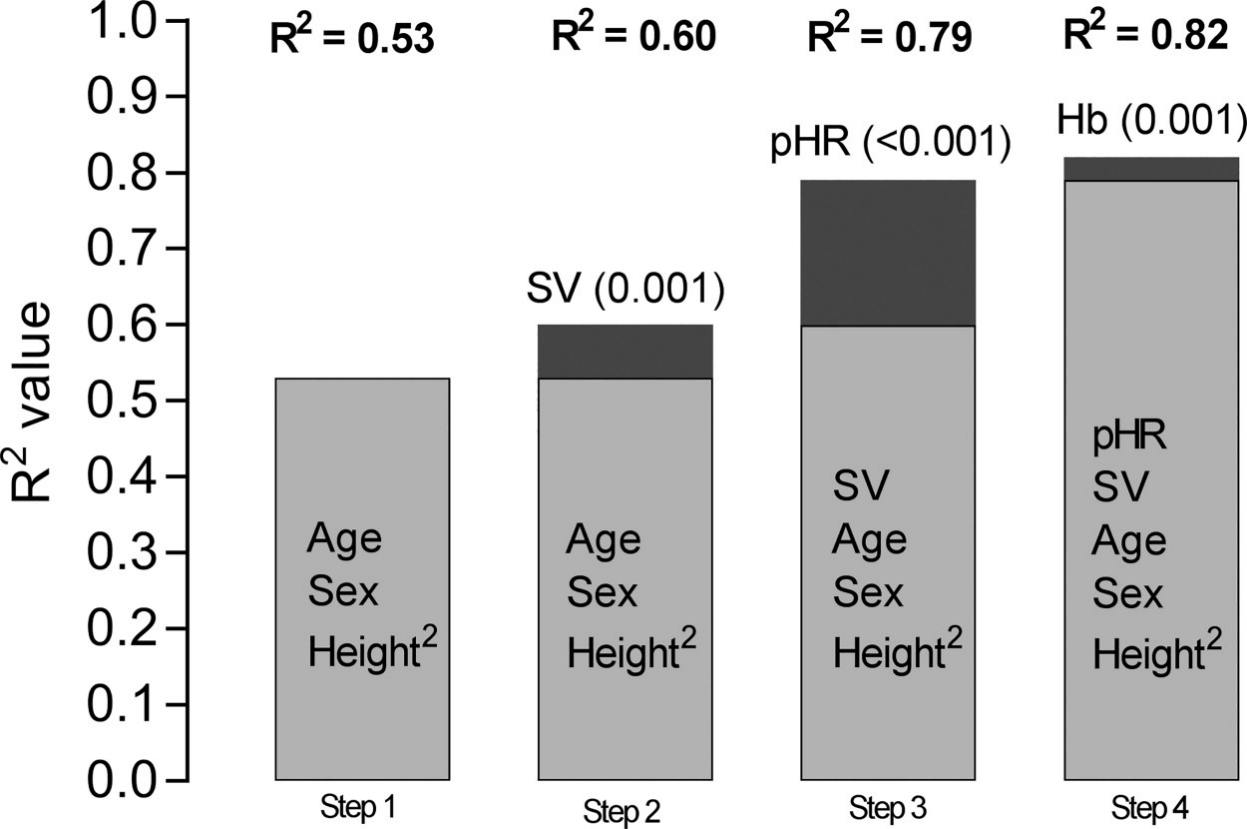
Stepwise increase in the explanatory value (R^2^) of variables determining exercise capacity in CKD 2–5 using manual forward regression analysis. Age, sex and height^2^ were included in the first step and, thereafter, one additional variable was added to the model to show the increase in R^2^ at each step. SV = stroke volume, pHR = peak heart rate, Hb = haemoglobin level. Number of subjects = 90 because of missing values for one or more variable. P-values in parenthesis. P-values in step 1: < 0.01 for all variables. P-values in step 2: height^2^ 0.1, all other variables <0.01. P-values in step 3: age 0.7, height^2^ 0.02, all other variables < 0.01. P-values in step 4: age 0.6, sex 0.04, height^2^ 0.04, all other variables < 0.01.

Fig 1 shows the stepwise increase in R^2^ after adding significant physiological variables from the multiple regression model shown in Table 5 in CKD patients. Adding the systemic oxygen delivery variables (SV, peak HR and haemoglobin level) individually to the model with age, sex and height^2^ produced an R^2^ of 0.83; peak HR made the strongest contribution to R^2^. Age was no longer a significant determinant when peak HR was included in the model.

A separate multiple regression analysis with age, sex, height^2^, SV, peak HR and haemoglobin level in patients not taking beta-blockers (n = 65) rendered an R^2^ of 0.81. Peak HR, SV and haemoglobin level still explained the significant variation in peak workload (p < 0.001, p < 0.001 and p = 0.007, respectively)

## Discussion

In mild to severe non-dialysis CKD, we found that aerobic ExCap, peak HR, haemoglobin level and the estimated peak systemic oxygen delivery decreased gradually with increasing disease severity. ExCap was strongly associated with the systemic oxygen delivery factors SV, peak HR and haemoglobin level. These factors, along with age, sex and height^2^, explained 83% of the individual variation in aerobic ExCap in the current CKD sample and peak HR was the main determinant of aerobic ExCap. The peripheral factors handgrip strength and vascular stiffness differed significantly only between CKD 4–5 compared to controls and LBM did not differ between groups. Moreover, after inclusion of the systemic oxygen delivery variables, these peripheral factors did not provide further explanation of the variance in ExCap and seem to be of less importance for aerobic ExCap in this group of CKD patients.

In CKD but not in healthy controls; peak HR, HR reserve, haemoglobin level and vascular stiffness were related to aerobic ExCap after adjusting for age, gender and height (Table 4). Reduced peak HR, as was seen already in mild-moderate CKD in our study, is a known feature of non-dialysis CKD [4, 37, 38]. Chinnappa et al reported that HR reserve was an independent determinant of aerobic ExCap in a male CKD population [4] which, to the best of our knowledge, is the only previous study in non-dialysis CKD that has reported a direct association between peak HR and ExCap. Our study included both men and women and shows that adding peak HR to a regression model with multiple determinants accounted for a substantial increase in R^2^ for peak workload in these CKD patients (Fig 1). Together with a high RC value for peak HR (Table 5), this suggests that chronotropic incompetence may be the single most important factor influencing ExCap in our non-dialysis CKD population. Our separate statistical analyses of CKD patients not taking beta-blockers showed that peak HR remained a strong determinant of ExCap, which suggests that rate-limiting medication was not the major reason for our findings. The rating of perceived exertion was equally high in the three study groups, indicating that most of the exercisers performed the test until exhaustion and maximal HR. A low effort does therefore not explain the observed decrease in peak HR in our study. In fact, the mechanisms responsible for the reduction in peak HR in CKD patients are unknown and surprisingly unexplored. It may involve both cardiac autonomic insufficiency [39] and peripheral factors (e.g., low muscle mass and/or mitochondrial dysfunction), both of which may reduce oxygen utilization and thus reduce peak HR, as has been proposed in heart failure [40]. We did not, however, find a significant decline in handgrip strength or lean mass in our CKD 2–3 group despite a significant decline in peak HR. The decline in aerobic ExCap in healthy individuals is primarily related to an age-related decline in peak HR [41]. CKD has been linked to a premature aging process [42], but it is unknown whether reduced peak HR is a characteristic of an accelerated aging process or whether other CKD factors cause chronotropic insufficiency. All regression models were adjusted for age, but age was no longer significant when peak HR was included. This suggests that, in CKD patients, peak HR is more strongly related to ExCap than to age. Peak HR has been shown to increase soon after renal transplantation, along with an increase in VO_2_max [43], which suggests that the process causing the reduced peak HR may be reversible. Our results also show that HR reserve is even more strongly associated with ExCap than is peak HR because HR reserve was the variable with the highest R^2^ when tested as a single determinant. This is expected because HR reserve is a composite measurement of both resting HR and peak HR, and indicates to a large extent the increase in systemic oxygen delivery during exercise.

In the multiple linear regression analyses, resting SV independently influenced aerobic ExCap in both the CKD patients and controls. In contrast to the gradual decrease in peak HR and haemoglobin level, resting SV did not differ significantly between any of the groups. This is consistent with previous studies in CKD that have compared CKD to healthy subjects [2, 4, 44]. To some extent this finding could be explained by effects of beta-blockers [4]. Peak SV was not assessed in our study but given the reduced ExCap in CKD it is likely that, as in elderly patients with cardiac diastolic dysfunction, a compensatory increase in SV offset by a low peak HR was not present [45, 46] Although our measurement of SV in the supine position is a close estimate of peak SV [35, 36], one cannot exclude a blunted SV response to exercise in some patients as the difference between peak exercise and rest was not assessed.

Haemoglobin level, which reflects arterial oxygen content and thereby the capacity for systemic oxygen delivery, decreased gradually in parallel to the gradual decrease in ExCap in the comparison between controls and the CKD 2–3 and CKD 4–5 subgroups. The relationship between reduced haemoglobin level and decreased aerobic ExCap is well known in moderate to severe CKD [16, 44, 47, 48] and our study shows that haemoglobin level is also important for ExCap in patients with mild to moderate CKD. Erythropoietin therapy has been shown to increase ExCap in CKD [15, 16, 47] although not as much as would be expected from the increase in haemoglobin concentration. This may be explained by peripheral factors such as a reduced oxygen conductance from the muscle capillaries to mitochondria and a thickened capillary endothelium, as shown in individuals on dialysis [49, 50], though yet not explored in non-dialysis CKD.

Pulse wave velocity has previously been found to be an independent determinant of aerobic ExCap in non-dialysis CKD [5] and in a population consisting of both non-dialysis and dialysis-dependent CKD [13]. Our measurement of vascular stiffness, Ep, was associated with aerobic ExCap in CKD 2–5 when tested as a single determinant, although in the multivariable analysis that included systemic oxygen delivery variables no independent association was found. The discrepancy may reflect the choice of variables included in multivariate analyses as well as the method used for measuring vascular stiffness.

We used handgrip strength, which is easily measured in clinical settings, a well-known indicator of overall upper body strength and associated with a risk estimate similar to that of quadriceps strength [51], as a proxy for skeletal muscle dysfunction. Handgrip strength is also a reliable measure of LBM [51], also demonstrated in the present study by a strong correlation between handgrip strength and LBM. Handgrip strength was not a significant determinant of ExCap in the multiple regression analysis when systemic oxygen delivery factors were included and was significantly lower only in CKD 4–5 compared with controls. Thus, it can be speculated that muscle function was not sufficiently reduced in our study population to have a significant influence on ExCap.

There are some limitations to the present study. The control group was not matched for medication, hypertension and diabetes, although we have taken into account beta-blocker use and diabetes in some analyses. We measured aerobic ExCap as peak workload and not VO_2_peak during exercise. However, given the linear relationship between exercise load and oxygen uptake during cycle ergometry [8] and the high ratings of perceived exertion indicating near-maximal effort, we consider our results to be valid measurements of aerobic ExCap in these CKD patients. Nonetheless, peak workload and VO_2_peak are not always interchangeable in severely diseased patients [52]. SV was assessed during supine rest and not during exercise. However, the increase from supine rest to maximal values for upright exercise is expected to be modest [35, 36]

In conclusion, this cross-sectional study demonstrates that aerobic ExCap in non-dialysis CKD decreases gradually with increasing disease severity and that ExCap is associated mainly with the systemic oxygen delivery factors: resting SV, peak HR and haemoglobin level, of which peak HR was the most important. Neither muscle function and mass nor vascular stiffness were independent determinants of ExCap in this group of patients. Future studies should focus on the mechanisms behind a reduced peak HR in CKD.

## Supporting information

S1 File Data file. (XLSX)

## Abbreviations

BP: blood pressure
CKD: chronic kidney disease
E/é: early filling velocity/early diastolic myocardial velocity
Ep: pressure strain elastic modulus in the carotid artery
ExCap: exercise capacity
GFR: glomerular filtration rate
HR: heart rate
HR reserve: difference between peak HR and resting HR
LBM: lean body mass
LV: left ventricular
RC: regression coefficient
SV: stroke volume
SVI: SV indexed for body surface area
VO_2_max: maximal oxygen uptake
VO_2_peak: peak oxygen uptake
